# Ectopic insulin secretion by a large‐cell neuroendocrine carcinoma of the cervix

**DOI:** 10.1002/ccr3.3562

**Published:** 2020-11-20

**Authors:** Mawson Wang, Quinlan Vasey, Winny Varikatt, Mark Mclean

**Affiliations:** ^1^ Department of Endocrinology Blacktown Hospital Sydney NSW Australia; ^2^ Blacktown Clinical School School of Medicine Western Sydney University Sydney NSW Australia; ^3^ ICPMR (Institute of Clinical Pathology and Medical Research) Westmead Hospital Sydney NSW Australia; ^4^ Westmead Clinical School University of Sydney Medical School Sydney NSW Australia

**Keywords:** hypoglycemia, insulinoma, large‐cell carcinoma, neuroendocrine carcinoma, Neuroendocrine tumor, octreotide

## Abstract

In patients presenting with hyperinsulinemic hypoglycemia with a nonpancreatic neuroendocrine tumor, the diagnosis of an ectopic insulin‐secreting tumor should be considered, and investigated further with confirmatory insulin staining.

## Graphical abstract

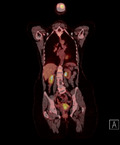

## 1 INTRODUCTION

Hypoglycemia in patients without diabetes mellitus is uncommon and is defined by plasma (or serum) glucose which is low enough to cause symptoms and/or signs, which may result in impaired neurological status.^1^ Further evaluation of hypoglycemia is required only in patients fulfilling Whipple's triad: (a) symptoms and/or signs consistent with hypoglycemia, (b) documented low plasma glucose concentration, and (c) relief of symptoms and/or signs after glucose concentration is raised.^1^ The approach to a hypoglycemic patient without diabetes mellitus should begin with a thorough history and physical examination to determine potential causes (Table 1).^1^, ^2^ During an episode of spontaneous hypoglycemia, measurement of plasma glucose, insulin, C‐peptide, proinsulin, and an oral hypoglycemic agent screen should be performed.^1^ When spontaneous hypoglycemia cannot be observed, it should be replicated by a 72‐hour fast or a mixed‐meal test. The presence of symptoms and/or signs with plasma glucose < 3 mmol/L, insulin ≥ 3 mIU/L, C‐peptide ≥ 0.2 nmol/L, and proinsulin ≥ 5 pmol/L indicates endogenous hyperinsulinemia.^1^

**Table 1:** Causes of hypoglycemia

Causes of hypoglycemia	
Drugs	Insulin or insulin secretagogue Alcohol
Critical illness	Organ failure, for example, cardiac, hepatic, or renal failure Sepsis
Hormone deficiency	Cortisol deficiency Glucagon and epinephrine deficiency in insulin‐deficient diabetes
Endogenous hyperinsulinemia	Islet cell tumor, for example, insulinoma Nonislet cell tumor, for example, bronchial carcinoid, gastrointestinal stroma tumor, paraganglioma Noninsulinoma pancreatogenous hypoglycemia Postgastric bypass hypoglycemia Insulin autoimmune hypoglycemia, for example, insulin or insulin receptor antibodies
Tumors without insulin secretion	Tumors producing insulin‐like growth factor 2, insulin‐like growth factor 1, somatostatin, glucagon‐like peptide 1.
Other	Accidental, surreptitious, or malicious hypoglycemia

After there is proven endogenous hyperinsulinemic hypoglycemia with negative screen for oral hypoglycemic agents and insulin antibodies, then further studies should be conducted to localize an insulinoma. This includes computed tomography (CT), magnetic resonance imaging, and transabdominal or endoscopic ultrasound. Treatment for hypoglycemia depends on the underlying etiology identified.

We present a case of an insulin‐secreting cervical neuroendocrine carcinoma causing significant hypoglycemia at 12 months after initial diagnosis.

## 2 CASE REPORT

A 62‐year‐old postmenopausal female presented to her general practitioner with postcoital bleeding. Her background history included two normal vaginal childbirths and tubal ligation. She was a lifelong nonsmoker, did not consume alcohol, and took no regular medications. Vaginal speculum examination, followed by hysteroscopy, demonstrated a tumor of the cervical lip. Papanicolaou smear and biopsy confirmed a human papilloma virus‐18‐positive cervical cancer. There were intermediate‐size malignant cells with oval, hyperchromatic, and overlapping nuclei, frequent mitosis, and necrosis. Tumor cells stained positively for p16, CD56, and synaptophysin, consistent with a neuroendocrine tumor.

**Figure 1:**
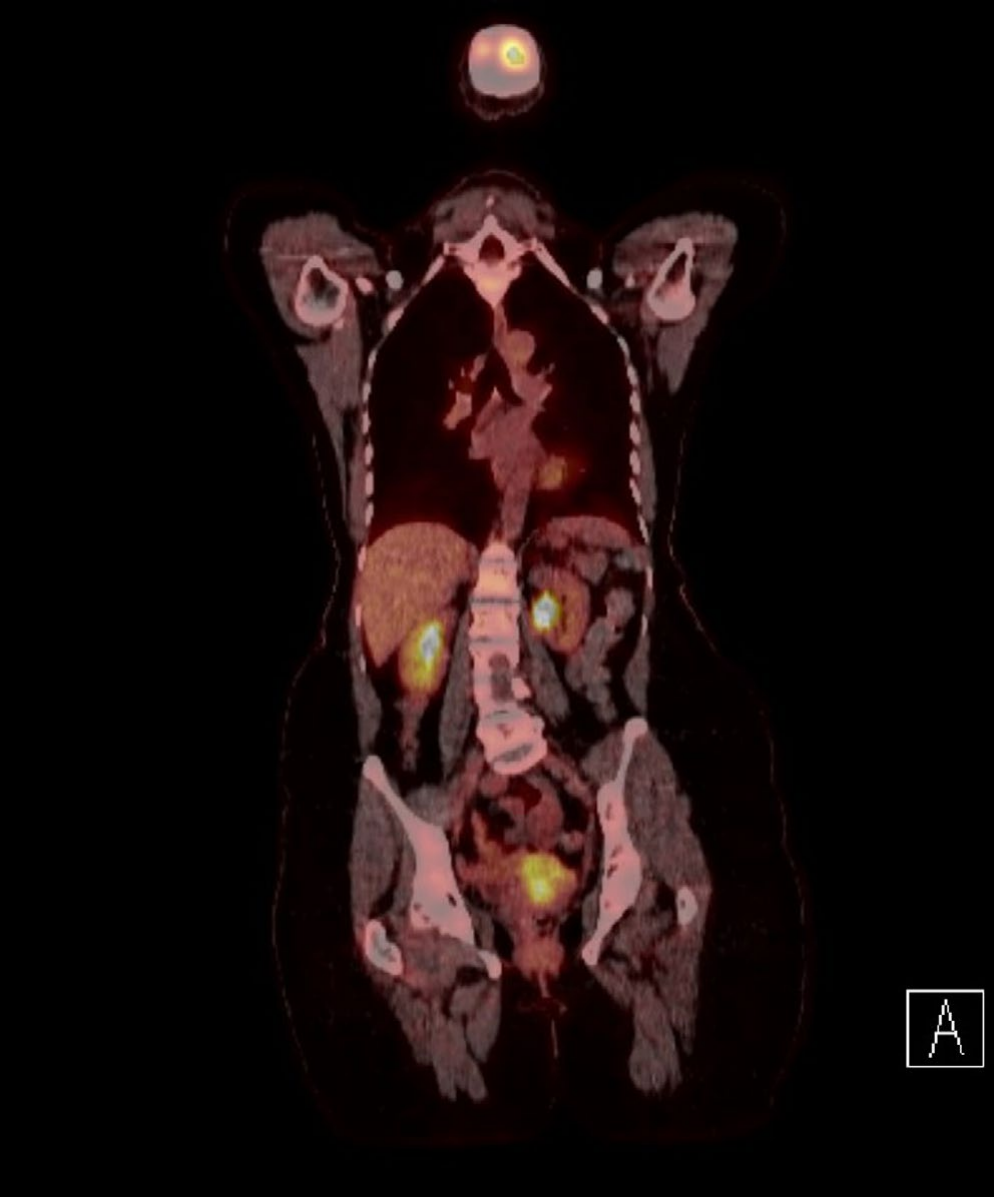
PET scan (prior to completion of therapy) showed partial metabolic response to treatment with significant decrease in extent and avidity of cervical lesion (SUV max 8.1, previously 13.5). No evidence of metabolically active local nodal or distant metastatic disease

**Figure 2:**
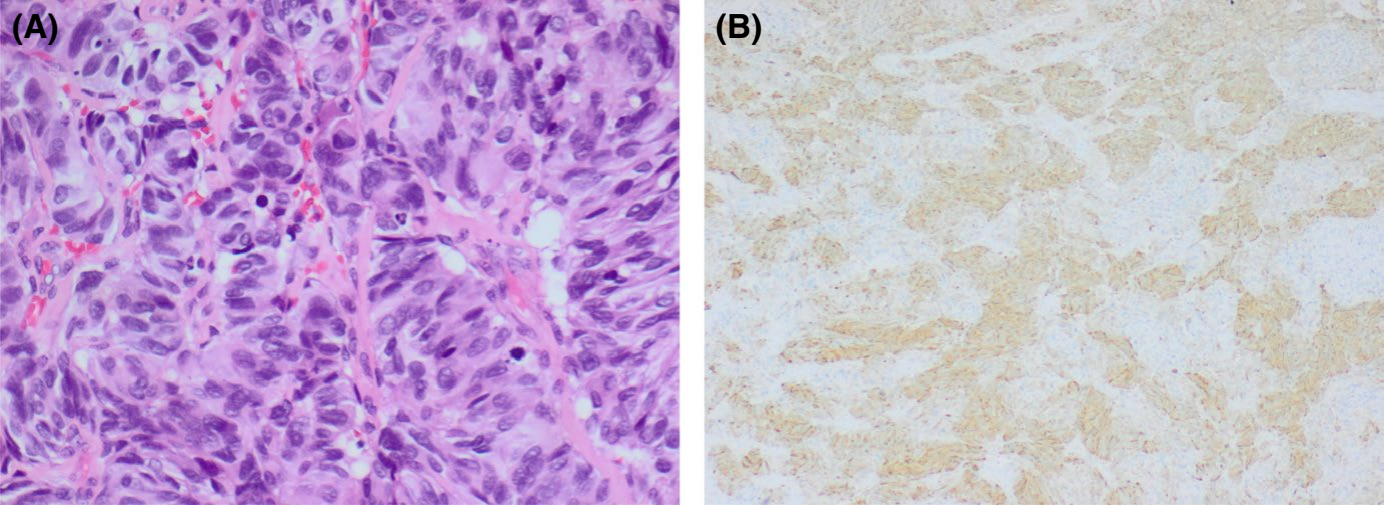
A, Hematoxylin‐and‐Eosin–stained section showing the large‐cell morphology and nested arrangement of cells (×400). B, Tumor cells display positive staining for Insulin (x200)

Ultrasound, CT, and 18F‐fluorodeoxyglucose positron emission tomography (FDG‐PET) confirmed a 3x4cm cervical mass without nodal or distant metastases, and a diagnosis of Stage IB2 neuroendocrine carcinoma of the cervix was made. She completed six cycles of carboplatin and etoposide with curative intent, followed by external beam radiotherapy and brachytherapy with a total biological equivalent dose of 90.8 Gray (Gy). A restaging FDG‐PET scan prior to completion of chemotherapy showed partial metabolic response in the cervix, but no local nodal or metastatic disease (Figure 1). Posttreatment biopsy demonstrated a tumor comprised of intermediate‐size neoplastic cells arranged in a nested pattern (Figure 2A). The tumor cells demonstrated neuroendocrine differentiation and a Ki‐67 proliferation index of 30%. The patient briefly travelled overseas to attend to personal matters before attending follow‐up at 6‐week postcompletion of all active treatment, when a PET scan was arranged.

**Figure 3:**
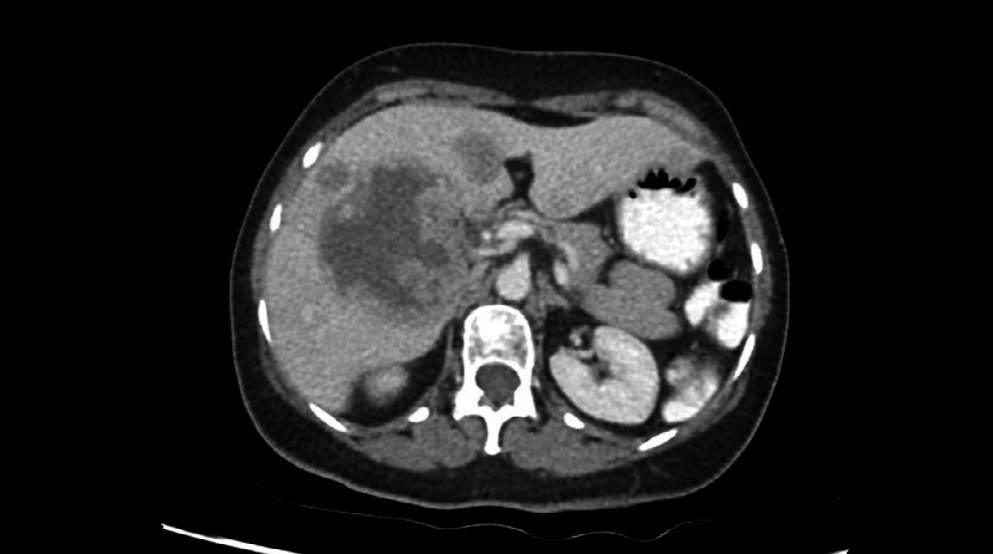
Multiple large liver metastases with central necrosis and peripheral enhancement, with largest lesion in Segment V measuring up to 7.5x6.2cm

At 12‐month postdiagnosis and prior to the planned PET scan, the patient presented to the emergency department with an episode of symptomatic hypoglycemia (plasma glucose 1.7 mmol/L) occurring in the fasted state. She was not receiving insulin or insulin secretagogues such as sulfonylureas. She exhibited sinus tachycardia to 120 bpm, sweating and tremor. Symptoms were promptly corrected by administration of glucose, fulfilling Whipple's triad. The patient also demonstrated marked pitting edema of the lower limbs without evidence of cardiac failure. Biochemical studies demonstrated normal renal function (estimated glomerular filtration rate > 90) and electrolytes, but marked liver function derangement, hypoalbuminemia to 21 g/L (normal range 35‐50), normocytic anemia to 75 g/L (normal range 115‐165), and thrombocytopenia to 25 × 10^9^g/L (normal range 150‐400). During one episode of hypoglycemia, when plasma glucose was 2.2 mmol/L, plasma C‐peptide was 2.33 nmol/L (normal range 0.26‐1.73) and plasma insulin 19mIU/L (normal range < 9). Chest X‐ray revealed widespread cannonball pulmonary metastases, and computed tomography revealed hepatic metastases (Figure 3), persistence of the cervical mass, and no evidence of a pancreatic lesion. Retrospective review of the cervical biopsy with further immunohistochemistry revealed positive staining for insulin and negative staining for glucagon confirming that her symptoms were due to an insulin‐secreting neuroendocrine carcinoma (NEC) (Figure 2B).

Initial treatment with intravenous boluses of 50% dextrose, maintenance 10% dextrose infusion, and intravenous hydrocortisone 100 mg QID were insufficient to maintain normoglycemia. Based on suspicions of an insulin‐secreting neuroendocrine carcinoma, the subcutaneous somatostatin analogue octreotide was commenced at 200 mcg q8hrly. This allowed the cessation of intravenous dextrose and hydrocortisone and clinically significant improvement in hypoglycemia (BSL between 4 and 10 mmol/L). Unfortunately, the metabolic panel was incomplete, as C‐peptide and insulin levels were not repeated after clinical interventions. Further acute hypoglycemic events were managed with oral glucose and subcutaneous glucagon 1 mg. In view of progressing aggressive malignancy and poor performance status, a palliative approach to management was decided. The patient died on day 20 of admission.

## 3 METHODS

Plasma insulin was measured using a one‐step chemiluminescent microparticle immunoassay, while plasma C‐peptide was measured using a two‐step chemiluminescent microparticle immunoassay (Abbott).

## 4 DISCUSSION

Neuroendocrine neoplasms (NENs) are malignancies that arise from neuroendocrine cells and may have the ability to produce and secrete peptide hormones. They typically originate in lung, gastrointestinal tract, or pancreas. NENs of the uterine cervix are rare and account for 0.9%‐1.5% of all cervical cancers.^3^ Recent updates to the classification of NEN, which emphasize tumor grade as opposed to anatomical origin, distinguish low‐grade neuroendocrine tumors from high‐grade NECs.^4^ Large‐cell NECs, as in this case, are less common than small‐cell NECs and are characterized morphologically by cells which are organized in organoid or trabecular patterns, with abundant cytoplasm, large nuclei with prominent nucleoli and high mitotic rate. Large‐cell NECs usually have relatively lower Ki‐67 proliferation index compared to small‐cell NECs which always demonstrate >90% proliferation index. Diagnosis is confirmed by positive immunohistochemistry for neuroendocrine markers (synaptophysin, CD56, and chromogranin).

Staging is determined by the International Federation of Gynecology and Obstetrics (FIGO) system.^5^ Our patient at the time of diagnosis had a 3 × 4 cm primary lesion of the uterine cervix without nodal or distant metastases detected by PET. In conjunction with tissue morphology and immunohistochemistry, this supports a diagnosis of a Stage 1B2 large‐cell neuroendocrine carcinoma of the cervix.

Due to the rarity of cervical NECs, no randomized controlled trials have been undertaken to guide management. Instead, treatment is informed by retrospective studies and treatment approaches extrapolated from the treatment of NEN arising from other organs such as small‐cell lung cancers. Typically, a multi‐modal approach is utilized involving radical surgery, radiotherapy, and chemotherapy involving etoposide and either carboplatin or cisplatin. Despite therapy, prognosis for cervical NECs remains poor, with a 5‐year survival of 36%.^6^

Nonislet cell tumors secreting insulin are infrequently reported, and comprise 1%‐2% of insulinomas,^7^ most commonly arising in peripancreatic or periduodenal regions. Ectopic insulin secretion has been reported in pheochromocytomas, and NENs of the kidney, liver, ovary, lung, and cervix.^7^ Positive insulin immunohistochemistry in the tumor, in combination with elevated C‐peptide and insulin levels, suggests that the insulin‐secreting tumor was the etiology for the patient's hypoglycemia. Interestingly, there had been no previous history of symptomatic hypoglycemia in the year since diagnosis and it is likely that declining hepatic gluconeogenesis due to an increasing metastatic tumor burden was a contributing factor in the pathophysiology. We are aware of only two previously reported cases^8^, ^9^ of insulin‐induced hypoglycemia originating from a cervical NEN, one of which was small cell, and the other a squamous cell carcinoma, although notably large‐cell NECs were frequently underrecognized and misdiagnosed as squamous cell carcinomas at the time of its report.^10^ In one patient, octreotide offered minimal clinical improvement and she required diazoxide and intravenous glucose.^8^ While resection of insulinoma is treatment of choice, medical therapy is considered for unresectable metastatic disease or poor surgical candidates. Diazoxide inhibits β‐cell insulin release and enhances glycogenolysis, however was not used in our patient due to her generalized edema and the propensity for diazoxide to cause fluid retention.^11^ Octreotide binds to somatostatin receptor type 2 and inhibits secretion of insulin, among other hormones.^11^ Phenytoin and verapamil also inhibit insulin release and have been used with varied success.^11^ Endoscopic ultrasound‐guided ethanol ablation and CT‐guided radiofrequency ablation of pancreatic insulinomas are minimally invasive procedures also considered in poor surgical candidates.^12^, ^13^

## 5 CONCLUSION

Ectopic insulin‐secreting neuroendocrine tumors are exceedingly rare, but should be considered as a differential diagnosis for hyperinsulinemic hypoglycemia. This may be confirmed through insulin staining on the tumor specimen. Medical therapy such as diazoxide and octreotide attempt to inhibit β‐cell insulin release, with mixed success.

## CONFLICT OF INTEREST

None declared.

## AUTHOR CONTRIBUTIONS

MW: was involved in the management of the case and wrote the manuscript; QV: assisted in the writing of the manuscript; WV: was involved in the immunohistochemistry of the specimen and assisted in the writing of the manuscript; MM: was involved in the management of the case and editing of the manuscript.

## ETHICAL STATEMENT

Ethics approval was not sought as this is a case report and all possible efforts were made to maintain patient anonymity.

## Data Availability

The datasets generated and/or analyzed during the current study are available from the corresponding author upon reasonable request.
